# Unilateral extravesical ureteral reimplantation via inguinal incision for the correction of vesicoureteral reflux: a 10-year experience

**DOI:** 10.1590/S1677-5538.IBJU.2016.0179

**Published:** 2017

**Authors:** Michael Yap, Unwanabong Nseyo, Hena Din, Madhu Alagiri

**Affiliations:** 1 Rady Children's Hospital, San Diego, CA, USA

**Keywords:** Urinary Incontinence, Minimally Invasive Surgical Procedures, Vesico-Ureteral Reflux

## Abstract

**Introduction and Objective:**

Multiple options exist for the surgical management of vesicoureteral reflux (VUR). We report on our 10-year experience using the inguinal approach to extravesical ureteral reimplantation (EVR).

**Materials and Methods:**

Patient characteristics of age, gender, and reflux grade were obtained and outcomes of operative time, hospital stay, and radiographic resolution were assessed.

**Results:**

71 girls and 20 boys with a mean age of 74 months (range 14-164) underwent inguinal EVR via a 3.5-cm inguinal mini-incision. Mean follow up was 10.9 months (range 0.4-69.7). Average grade of reflux was 2.80. Average operative time was 91 minutes (range 51-268). The procedure was successful in 87 of 91 patients (95.6%). The 3 cases of reflux that persisted were all grade 1 and managed expectantly. Contralateral reflux developed in 9 cases, all of which resolved after treatment with either Deflux or ureteral reimplant. There were 4 case of urinary retention that resolved after a brief period of CIC or indwelling catheterization. There were no cases of ureteral obstruction. Most patients were discharged on post-operative day 1 (85/91) and no hospitalization extended beyond 3 days.

**Conclusions:**

The inguinal approach to extravesical ureteral reimplantation should be considered as a potentially minimally invasive alternative to endoscopic and robotic treatment of VUR with a success rate more comparable to traditional open approaches. We feel it is the method of choice in cases of unilateral VUR requiring surgical correction.

## INTRODUCTION

Multiple options exist for the surgical management of vesicoureteral reflux (VUR). Since Lich (1) and Gregoir et al. (2) first popularized extravesical reimplantation (EVR) in the early 1960s, various refinements and modifications have been described. In 2002, Chen et al. reported on EVR performed through an inguinal approach rather than through the standard Pfannenstiel incision (3). They showed that inguinal EVR was safe, effective, and associated with a shorter hospital stay, shorter operative time, and less postoperative pain when compared to both standard EVR and intravesical reimplantation (4). We now report on our 10-year experience with inguinal EVR for the management of VUR. To our knowledge, this is the largest single series to report on extravesical ureteral reimplantation through an inguinal incision. We hypothesized that unilateral inguinal EVR would remain a safe and effective procedure with opportune cosmesis when evaluated within a large cohort.

## MATERIALS AND METHODS

**Figure 1:**
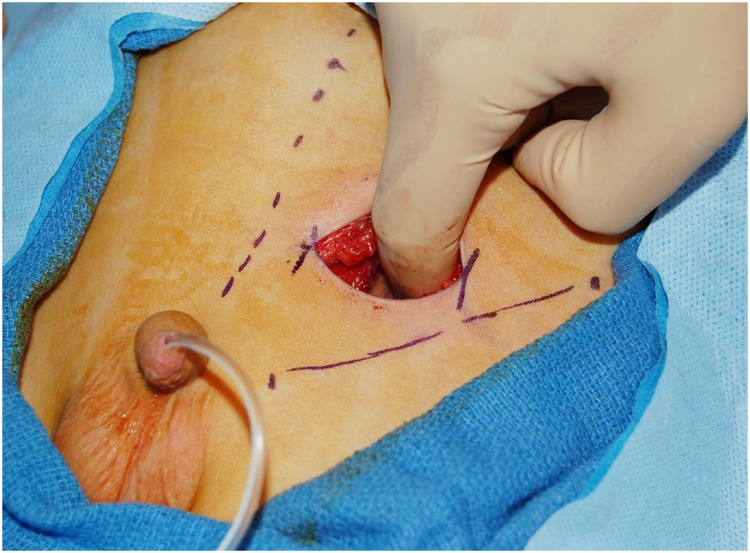
Intraoperative example of inguinal incision.

After obtaining IRB approval, we retrospectively reviewed the charts of 157 patients who underwent unilateral inguinal EVR between July 2002 and October 2012. All surgeries were performed by a single surgeon. Average inguinal incision was 3.5cm in length (Figure-1). Baseline characteristics assessed included patient age at the time of surgery, gender, presence of duplicated system, and indication for surgery. We also reviewed length of hospitalization and operative time. The primary objective of our study was to determine surgical success, which was defined as the absence of reflux in the ipsilateral ureter on postoperative voiding cystourethrogram (VCUG). Secondary objectives included determining the presence of de novo contralateral VUR, postoperative urinary retention, and postoperative urinary obstruction. Urinary retention was defined as the need for Foley catheter replacement or clean intermittent catheterization (CIC) postoperatively. Urinary obstruction was defined as postoperative hydronephrosis requiring intervention. Patients were excluded from the study if they had secondary VUR, lack of preoperative or postoperative VCUG, incomplete records, or if surgery was performed through a Pfannenstiel incision.

## RESULTS

**Figure 2:**
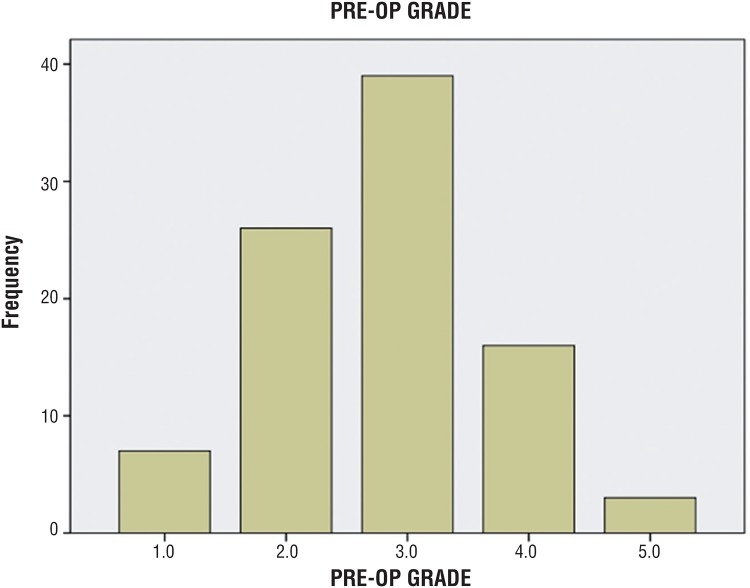
Distribution of preoperative reflux grades.

A total of 71 girls and 20 boys met study criteria for a total of 91 patients in the cohort. Patient demographics are presented in Table-1. Mean patient age was 74 months (range: 14-164 months), with females being significantly older than males at the time of surgery (50.3 months vs. 30.6 months, p=0.006). Average grade of reflux was 2.80, with the distribution of grades shown in Figure-2. Indication for surgery in the majority of patients was persistent asymptomatic VUR and parental preference (62 of 91 patients). The remainder of patients underwent surgery for renal scarring, parental preference, or recurrent UTI. Eight patients had previously undergone ipsilateral surgery for VUR. Seven patients had prior subureteric injection of Deflux^®^ and one patient had prior ureteroneocystostomy. Common sheath reimplantation for duplicated collecting systems was performed in 14 patients. 88 patients underwent inguinal EVR alone with no other concomitant procedures. Average operative time in these patients was 91 minutes (range: 51-268 minutes). No intraoperative complications occurred. Average hospital stay was 1.08 days (range: 1-3 days), with the majority of patients discharged on postoperative day 1 (85 of 91 patients). Follow-up ranged from 0.4 to 69.7 months, with a mean of 10.9 months.

**Table 1:** Patient characteristics.

Total Number of Patients		91	
**Gender, n (%)**			
	Male	20 (22.0)	
	Female	71 (78.0)	
Mean (range) age, months		74.4 (14-164)	p = 0.006
	Male	55.8 (15-164)	
	Female	79.7 (14-159)	
**Indication for surgery, n (%)**			
Recurrent UTI		8 (8.8)	
Persistent Asymptomatic VUR		62 (68.1)	
Parental Preference		6 (6.6)	
Renal Scarring		15 (16.5)	
Duplicated System, n (%)		14 (15.4)	
Prior Ipsilateral VUR Surgery, n (%)		8 (8.8)	
	Deflux	7 (7.7)	
	Reimplant	1 (1.1)	

The overall success rate in our series was 95.6% (87 of 91 patients) (Table-2). Persistent reflux was grade 1 in all four cases and none required intervention in the follow-up period. Follow-up VCUG was not performed in any patient to assess for resolution of persistent reflux.

**Table 2:** Outcomes.

Resolved reflux, n (%)	87 (95.6)
Contralateral de novo reflux, n (%)	9 (9.9)
Urinary retention, n (%)	4 (4.4)
Urinary obstruction, n (%)	0 (0)
Operative time, mean (range) min	91 (51-268)
Hospital stay, mean (range) days	1.08 (1-3)

Nine patients (9.9%) developed de novo contralateral reflux postoperatively (Table-3). Of the patients with de novo reflux, four (44%) had a history of reflux that had resolved prior to surgery. Eight patients had resolution of contralateral reflux following either Deflux^®^ or formal reimplantation. One patient had spontaneous resolution of de novo contralateral reflux. One patient was lost to follow-up.

**Table 3:** Characteristics of patients with *de novo* contralateral VUR.

Patient	Age (months)	Gender	Preoperative VUR Grade	Contralateral VUR Grade	History of Bilateral VUR	Intervention (Resolution)	Resolution
1	50.3	Female	4	1	No	Deflux^®^	Yes
2	50.4	Female	2	2	No	Lost to follow-up	Lost to follow-up
3	42.5	Female	4	2	No	Observation	Yes
4	54.4	Female	3	2	Yes	Reimplantation	Yes
5	112.5	Female	4	2	Yes	Reimplantation	Yes
6	112.5	Female	4	2	No	Deflux^®^	Yes
7	71.5	Female	3	2	Yes	Deflux^®^	Yes
8	72.5	Female	4	3	Yes	Reimplantation	Yes
9	86.0	Female	3	3	No	Deflux^®^	Yes

Urinary retention developed in 4 children (4.4%) and was transient in all cases (Table-4). Three of these four patients resumed normal voiding after CIC for one day or less. The other patient failed multiple voiding trials and ultimately required prolonged indwelling Foley drainage. This patient had a significant history of voiding dysfunction and ultimately resumed spontaneous voiding 20 days after surgery. Ureteral obstruction did not occur in any patient.

**Table 4:** Characteristics of patients with postoperative urinary retention.

Patient	Age (months)	Gender	Intervention	Duration (days)	Resolution
1	30.6	Male	Indwelling Foley	20	Yes
2	89.9	Female	CIC	1	Yes
3	68.2	Female	CIC	1	Yes
4	77.8	Female	CIC	1	Yes

**CIC =** Clean intermittent catheterization

## DISCUSSION

Ureteral reimplantation is a definitive surgical therapy with a high success rate for eliminating VUR. The Lich-Gregoir extravesical reimplantation technique was first introduced in the early 1960s as an alterative to traditional transvesical repair and has since been shown to be effective in greater than 90% to 95% of patients (5-7). This technique is thought by many to be less morbid than intravesical techniques that require cystotomy and direct urothelial manipulation (8). In a prospective, randomized trial comparing open intravesical reimplantation to EVR for unilateral reflux, Schwenter et al. (6) showed that the extravesical approach resulted in shorter operative time, avoidance of gross hematuria, and less postoperative pain and bladder spasms (8).

Traditionally, extravesical reimplantation has been performed through a standard Pfannenstiel incision. In 2002, Chen et al. (3) were the first to describe EVR through an inguinal approach, which they successfully and safely performed in the outpatient setting (3). They later compared inguinal EVR to conventional EVR through a Pfannenstiel incision and reported decreased operative time, shorter hospital stay, and reduced postoperative analgesic requirements with the inguinal approach (4).

The ability to perform inguinal EVR in the outpatient setting potentially marginalizes the benefits of traditional minimally invasive techniques, such as endoscopic injection of dextranomer/hyaluronic acid (Deflux^®^) and robotic ureteral reimplantation. Transurethral injection of Deflux^®^ offers a less invasive alternative to open surgery but at the expense of lower success rates and questionable long-term durability (9-12). Additionally, when compared to outpatient EVR in unilateral cases of reflux, Deflux^®^ was been shown to be the more expensive of the two procedures (13).

**Figure 3:**
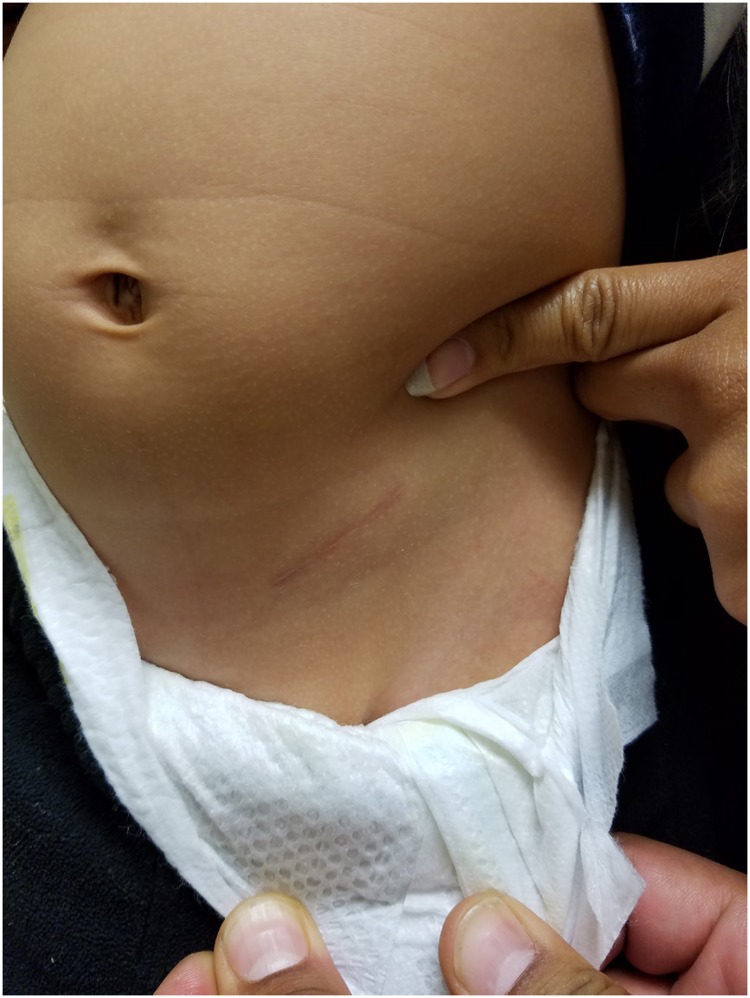
Inguinal incision 3 months postoperatively.

The use and applicability of robotic surgery for correction of VUR remains a highly debated topic. Advocates cite advantages of improved cosmesis, decreased pain, reduced hospital stay, and high success rates (14-16). A recent multi-institutional review by Grimsby et al. (15), however, showed a lower success rate, higher complication rate, and longer operative times when compared to open ureteral reimplantation (17). Long-term durability has also yet to be documented for robotic surgery and its use may ultimately be limited by higher costs, a steep learning curve, and limited accessibility (18). In regards to cosmesis, we maintain that a small inguinal incision, that can be concealed below the underwear or bathing suit line, provides a superior cosmetic result when compared with 2 to 4 abdominal scars associated with the robotic approach. This is supported by validated scar surveys in pyeloplasty patients which have shown that parents and patients prefer incisions that can be hidden over laparoscopic incisions that are more conspicuous (19). An example of our inguinal incision 3 months post-operatively is shown in Figure-3.

To our knowledge, we report on the largest single series experience with inguinal EVR for the management of VUR. In our 10-year cohort, we report a collective 95.6% success rate in treating 91 patients with unilateral inguinal EVR. This is comparable to success rates reported by prior studies describing their experiences with inguinal EVR (3, 4, 8, 20). Previously, Chen et al. (3) reported 1 failure in 89 patients and Schwenter et al. (6) reported a 100% success rate in 22 patients after inguinal EVR (3, 8). In 2011, Wiygul and Palmer (9) reported on their experience with inguinal EVR in 45 patients (20). Although postoperative VCUG was not routinely obtained in their study, the 3 patients in their series with febrile UTIs postoperatively did not have persistent reflux when VCUG was repeated.

Previous studies have looked at potential risk factors for persistent VUR after open reimplantation and identified male gender, high preoperative VUR grade, dysfunctional voiding, preoperative hydronephrosis, ureteral tapering, and younger age as features that might increase the chances of failed repair (21-24). The small number of patients with persistent VUR in our series limited our ability to detect associations or to perform large multivariate analyses; however, we did note that all 4 patients with persistent reflux in our study had a prior history of contralateral reflux that spontaneously resolved prior to surgery. While we did not repeat VCUG in any patient with persistent VUR, studies suggest that the natural history is eventual resolution (21). Hubert et al. (25) reported persistent VUR in 27.8% of their cohort, with spontaneous resolution in all cases that were grade 1. In our series, all 4 cases of persistent VUR were grade 1 (24).

De novo contralateral reflux developed in 9.9% of our cohort. Studies have previously identified younger age, smaller than expected bladder capacity, and history of preoperatively resolved contralateral VUR as risk factors for de novo contralateral VUR after open unilateral reimplantation (24, 26, 27). In our series, we noted that nearly 50% of patients with de novo contralateral VUR had a history of resolved contralateral VUR, although no statistical analysis was performed.

Hubert et al. (25) evaluated the natural history of contralateral VUR in 39 patients and reported a 78% rate of spontaneous resolution at a median of 23 months (24). In our series, all 7 patients who underwent intervention for de novo contralateral reflux were asymptomatic at the time of surgery; accordingly, it may have been reasonable to manage these patients with observation alone. In patients who are asymptomatic but whose parents prefer surgery, however, Deflux^®^ seems to be a good first option.

There were 14 duplicated systems in our cohort with successful correction of VUR in 13 of these patients (92.9%). Radojicic et al. (28) previously described their experience using inguinal EVR for reflux in duplicated ureters and reported successful repair in all 14 patients in their series (25). These findings suggest that inguinal EVR can be successfully used for correcting reflux in both straightforward and complex anatomy.

While postoperative urinary retention remains a feared complication following EVR, particularly in bilateral cases, it seems to be less of a concern after unilateral procedures (6, 29, 30). In our study, only 4 of 91 patients (4.4%) developed post-operative urinary retention, which was transient in all cases. While transvesical techniques may be more appropriate for bilateral cases of VUR, we believe our findings support the use of EVR as the technique of choice in open reimplantation for unilateral reflux.

Ureteral obstruction is a rare complication after extravesical reimplantation and no patients in our series experienced urinary obstruction requiring intervention.

In our study, a total of 7 patients were excluded from the analysis due to the absence of post-operative VCUG. All resulted of loss to follow-up. It is possible that these patients may have had follow-up care elsewhere, in which case we may have missed surgical failures and/or post-operative complications (i.e. urinary retention, ureteral obstruction, etc.). However, it is our belief that they these patients were actually more likely to have had a successful outcome and less likely to have experienced post-operative complications and therefore did not feel the need to follow-up. This would then have led to an underestimation of the efficacy of inguinal EVR and overestimation of its associated complications, such as urinary retention and ureteral obstruction.

## CONCLUSIONS

Ureteral reimplantation may be safely and successfully performed through an inguinal hernia incision by using the extravesical technique. In unilateral cases, postoperative urinary retention following inguinal EVR is rare. This approach avoids the adverse effects of entering the bladder and can offer an outpatient alternative to endoscopic therapy. This technique should be considered as a potentially minimally invasive alternative to endoscopic and robotic treatment of VUR with a success rate more comparable to traditional open approaches. Accordingly, we feel it is the method of choice in cases of unilateral VUR requiring surgical correction. Our study adds to the limited literature regarding use of inguinal EVR for the management of vesicoureteral reflux.
